# Poly[μ_2_-aqua-μ_4_-[1-(4-chloro­phen­yl)-4,4,4-tri­fluoro­butane-1,3-dionato]-potassium]

**DOI:** 10.1107/S1600536813017388

**Published:** 2013-06-29

**Authors:** João P. Martins, Cláudia C. Arranja, Abílio J. F. N. Sobral, Manuela Ramos Silva

**Affiliations:** a CEMDRX, Physics Department, University of Coimbra, P-3004-516 Coimbra, Portugal; b Chemistry Department, University of Coimbra, P-3004 Coimbra, Portugal

## Abstract

In the title compound, [K(C_10_H_5_ClO_2_F_3_)(H_2_O)]_*n*_, the two independent K^+^ ions are located on a twofold rotation axis. For each of the cations, the distorted cubic coordination environment is defined by two F and four O atoms of symmetry-related 1,4-chloro­phenyl-4,4,4-tri­fluoro­butane-1,3-dionate anions and by two O atoms of water mol­ecules. The μ_4_-bridging character of the anion and the μ_2_-bridging of the water mol­ecule lead to the formation of layers parallel to (100). The coordinating water mol­ecules are also involved in O—H⋯O hydrogen bonds that reinforce the mol­ecular cohesion within the layers, which are stacked along [100]. The β-diketonate anion is not planar, with an angle of 31.78 (10)° between the mean planes of the diketonate group and the chloro­phenyl ring.

## Related literature

For background to lanthanide complexes with diketonate ligands, see: Martín-Ramos *et al.* (2013*a* ▶,*b* ▶).
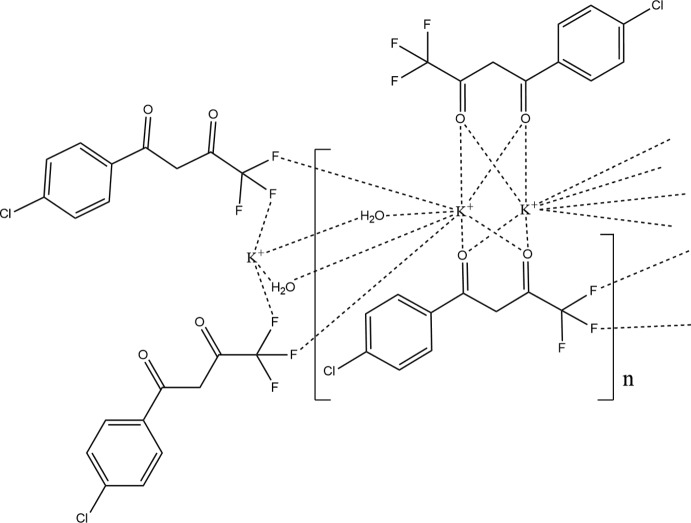


## Experimental

### 

#### Crystal data

[K(C_10_H_5_ClF_3_O_2_)(H_2_O)]
*M* *_r_* = 306.71
Monoclinic, 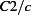

*a* = 30.164 (2) Å
*b* = 8.0739 (4) Å
*c* = 10.2696 (5) Å
β = 98.752 (2)°
*V* = 2471.9 (2) Å^3^
*Z* = 8
Mo *K*α radiation
μ = 0.68 mm^−1^
*T* = 293 K
0.20 × 0.11 × 0.08 mm

#### Data collection

Bruker APEX CCD area-detector diffractometer
Absorption correction: multi-scan (*SADABS*; Bruker, 2003 ▶) *T*_min_ = 0.830, *T*_max_ = 0.999
11396 measured reflections
2182 independent reflections
1559 reflections with *I* > 2σ(*I*)
*R*_int_ = 0.032

#### Refinement

*R*[*F* ^2^ > 2σ(*F* ^2^)] = 0.037
*wR*(*F* ^2^) = 0.097
*S* = 1.01
2182 reflections
170 parameters
H atoms treated by a mixture of independent and constrained refinement
Δρ_max_ = 0.19 e Å^−3^
Δρ_min_ = −0.20 e Å^−3^

### 

Data collection: *SMART* (Bruker, 2003 ▶); cell refinement: *SAINT* (Bruker, 2003 ▶); data reduction: *SAINT*; program(s) used to solve structure: *SHELXS97* (Sheldrick, 2008 ▶); program(s) used to refine structure: *SHELXL97* (Sheldrick, 2008 ▶); molecular graphics: *ORTEPII* (Johnson, 1976 ▶); software used to prepare material for publication: *SHELXL97*.

## Supplementary Material

Crystal structure: contains datablock(s) global, I. DOI: 10.1107/S1600536813017388/wm2752sup1.cif

Structure factors: contains datablock(s) I. DOI: 10.1107/S1600536813017388/wm2752Isup2.hkl

Additional supplementary materials:  crystallographic information; 3D view; checkCIF report

## Figures and Tables

**Table 1:** Hydrogen-bond geometry (Å, °)

*D*—H⋯*A*	*D*—H	H⋯*A*	*D*⋯*A*	*D*—H⋯*A*
O3—H1*W*⋯O2	0.82 (3)	1.90 (3)	2.709 (2)	173 (3)
O3—H2*W*⋯O1^i^	0.87 (3)	2.06 (3)	2.843 (3)	150 (2)

Symmetry code: (i) 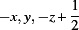.

**Fig. 1.**
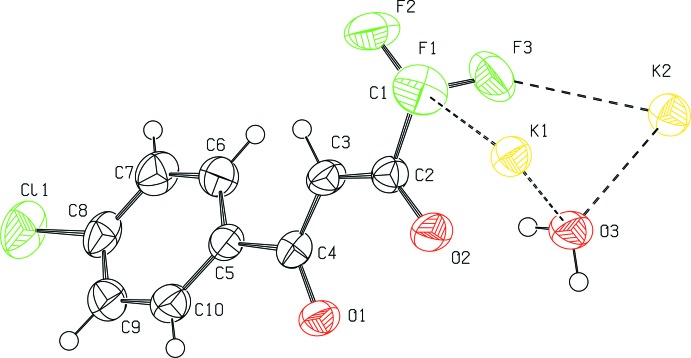
ORTEPII plot of the asymmetric unit of the title compound. Displacement ellipsoids are drawn at the 50% probability level.

**Fig. 2.**
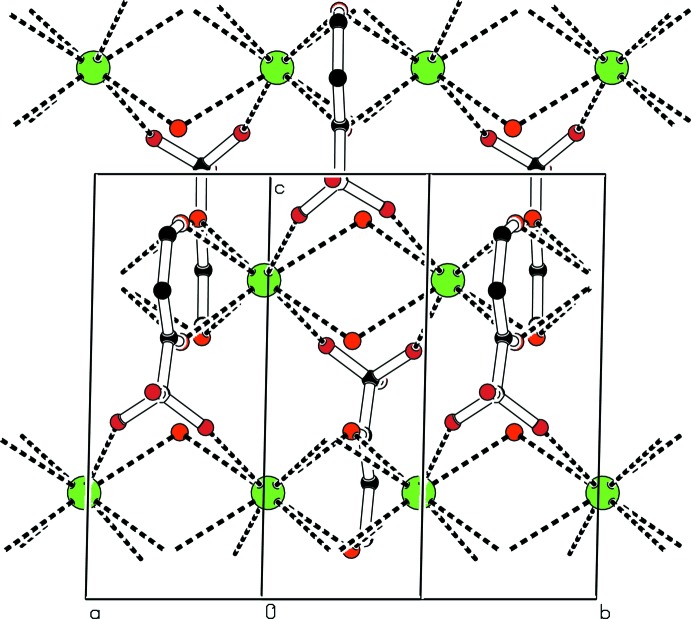
Packing of the components in the title structure. For clarity, the Cl and H atoms were omitted. Atoms C5 to C10 defining the benzene ring were also omitted.

**Fig. 3.**
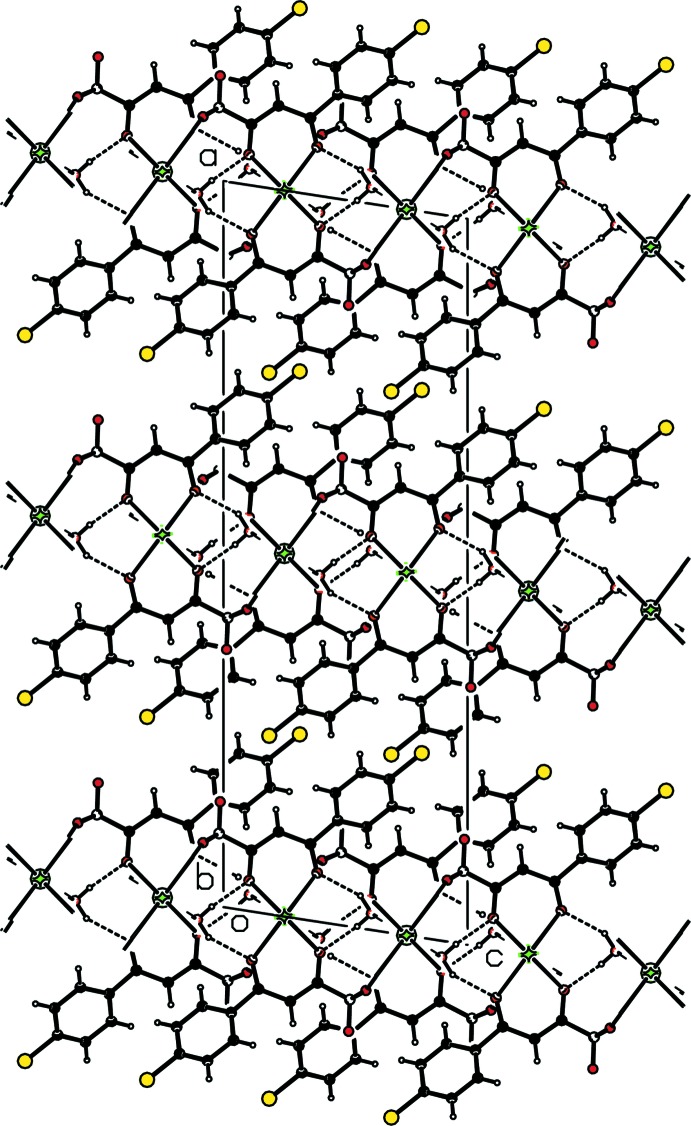
Packing of the components showing the hydrogen bonding interactions as dashed lines.

## supplementary crystallographic information

### Comment

The title compound, [K(C_10_H_5_ClO_2_F_3_)(H_2_O)], Fig. 1, was obtained serendipitously as part of a project to synthesize new lanthanide coordination complexes as potential emissive layers in organic light emitting diodes (OLEDs) (Martín-Ramos, 2013*a*,*b*).

The title compound contains two potassium ions, one 1,4-chlorophenyl-4,4,4-trifluoro-1,3-butanedionate anion and one coordinating water molecule in the asymmetric unit. Both potassium ions are situated on twofold rotation axes and are in the centres of distorted cubes, that are formed by two F and six O atoms. The cations are arranged in alternating chains along [010], Fig. 2, with K···K distances of 3.6379 (11) and 4.4360 (11) Å, respectively. The cations are bridged by two water molecules and one bis-monodentate CF_3_ group, as well as by four oxygen atoms of two β-diketonate groups. The chains are joined into layers parallel to (100) since each diketonate coordinates potassium ions from two adjacent chains. The β-diketonate ligand is not planar with an angle of 31.78 (10)° between the mean planes of the diketonate group and the chlorophenyl ring. Within the layers, there are hydrogen bonds between the coordinating water molecules and adjacent diketonate O atoms (Table 1, Fig. 3). The unit cell does not contain any residual solvent acessible voids.

### Experimental

Firstly, 0.5 mmol of europium(III) nitrate pentahydrate were dissolved in 20 ml of methanol followed by the addition of 0.9 ml of potassium methoxide. This solution was left to reflux at 353 K for 15 min. Secondly, 1.5 mmol of 1,14 chlorophenyl-4,4,4-trifluoro-1,3-butanedionate were dissolved in 15 ml of methanol and added to the main solution. After decanting the resulting solution, 0.5 mmol of bathophenanthroline were dissolved in 10 ml of methanol and added to the main solution. The main solution was then transferred from a volumetric balloon to a beaker covered with paraffin film and placed on a water bath at 303 K until complete evaporation was verified. Since from the evaporation process no crystals were obtained, all the material from this batch was dissolved in 25 ml of chloroform. A light orange powder was formed alongside with some transparent crystals. The powder was studied by X-ray powder diffraction and was proven to be amorphous; the transparent crystals were studied by single-crystal X-ray diffraction, and as a result, the title compound was revealed.

### Refinement

All hydrogen atoms bound to carbon atoms were placed at calculated positions and were treated as riding on the parent atoms with C—H = 0.93 Å (aromatic) and with *U*_iso_(H) = 1.2 *U*_eq_(C). The H atoms belonging to the water molecule were found in a difference electron density synthesis and subsequently refined with *U*_iso_(H)=1.2*U*_iso_(O).

### Crystal data

**Table:**

[K(C_10_H_5_ClF_3_O_2_)(H_2_O)]	*F*(000) = 1232
*M**_r_* = 306.71	*D*_x_ = 1.648 Mg m^−^^3^
Monoclinic, *C*2/*c*	Mo *K*α radiation, λ = 0.71073 Å
Hall symbol: -C 2yc	Cell parameters from 3474 reflections
*a* = 30.164 (2) Å	θ = 2.7–23.9°
*b* = 8.0739 (4) Å	µ = 0.68 mm^−^^1^
*c* = 10.2696 (5) Å	*T* = 293 K
β = 98.752 (2)°	Prism, colourless
*V* = 2471.9 (2) Å^3^	0.20 × 0.11 × 0.08 mm
*Z* = 8	

### Data collection

**Table:**

Bruker APEX CCD area-detector diffractometer	2182 independent reflections
Radiation source: fine-focus sealed tube	1559 reflections with *I* > 2σ(*I*)
Graphite monochromator	*R*_int_ = 0.032
φ and ω scans	θ_max_ = 25.8°, θ_min_ = 2.6°
Absorption correction: multi-scan (*SADABS*; Bruker, 2003)	*h* = −35→36
*T*_min_ = 0.830, *T*_max_ = 0.999	*k* = −9→9
11396 measured reflections	*l* = −12→11

### Refinement

**Table:**

Refinement on *F*^2^	Primary atom site location: structure-invariant direct methods
Least-squares matrix: full	Secondary atom site location: difference Fourier map
*R*[*F*^2^ > 2σ(*F*^2^)] = 0.037	Hydrogen site location: inferred from neighbouring sites
*wR*(*F*^2^) = 0.097	H atoms treated by a mixture of independent and constrained refinement
*S* = 1.01	*w* = 1/[σ^2^(*F*_o_^2^) + (0.0532*P*)^2^ + 0.5088*P*] where *P* = (*F*_o_^2^ + 2*F*_c_^2^)/3
2182 reflections	(Δ/σ)_max_ < 0.001
170 parameters	Δρ_max_ = 0.19 e Å^−^^3^
0 restraints	Δρ_min_ = −0.20 e Å^−^^3^

### Special details

**Table:**

Geometry. All e.s.d.'s (except the e.s.d. in the dihedral angle between two l.s. planes) are estimated using the full covariance matrix. The cell e.s.d.'s are taken into account individually in the estimation of e.s.d.'s in distances, angles and torsion angles; correlations between e.s.d.'s in cell parameters are only used when they are defined by crystal symmetry. An approximate (isotropic) treatment of cell e.s.d.'s is used for estimating e.s.d.'s involving l.s. planes.
Refinement. Refinement of *F*^2^ against ALL reflections. The weighted *R*-factor *wR* and goodness of fit *S* are based on *F*^2^, conventional *R*-factors *R* are based on *F*, with *F* set to zero for negative *F*^2^. The threshold expression of *F*^2^ > σ(*F*^2^) is used only for calculating *R*-factors(gt) *etc*. and is not relevant to the choice of reflections for refinement. *R*-factors based on *F*^2^ are statistically about twice as large as those based on *F*, and *R*- factors based on ALL data will be even larger.

### Fractional atomic coordinates and isotropic or equivalent isotropic displacement parameters (Å^2^)

**Table:**

	*x*	*y*	*z*	*U*_iso_*/*U*_eq_	
K1	0.0000	−0.01267 (9)	0.7500	0.0468 (2)	
K2	0.0000	0.53676 (9)	0.7500	0.0505 (2)	
Cl1	−0.25418 (2)	0.31387 (12)	−0.31250 (7)	0.0774 (3)	
F1	−0.08518 (5)	0.13336 (19)	0.59626 (14)	0.0684 (5)	
F2	−0.14447 (5)	0.2619 (2)	0.51303 (15)	0.0765 (5)	
F3	−0.08456 (5)	0.3946 (2)	0.58351 (15)	0.0742 (5)	
O1	−0.06701 (5)	0.22988 (19)	0.11627 (16)	0.0462 (4)	
O2	−0.04203 (5)	0.2360 (2)	0.39484 (16)	0.0536 (5)	
O3	0.02269 (7)	0.2670 (2)	0.60726 (18)	0.0554 (5)	
H1W	0.0018 (10)	0.254 (3)	0.548 (3)	0.067*	
H2W	0.0446 (10)	0.267 (3)	0.561 (3)	0.067*	
C1	−0.10053 (8)	0.2594 (3)	0.5178 (2)	0.0463 (6)	
C2	−0.08424 (7)	0.2490 (3)	0.3853 (2)	0.0390 (6)	
C3	−0.11551 (7)	0.2554 (3)	0.2741 (2)	0.0433 (6)	
H3	−0.1455	0.2651	0.2848	0.052*	
C4	−0.10548 (7)	0.2484 (3)	0.1436 (2)	0.0388 (6)	
C5	−0.14351 (7)	0.2644 (3)	0.0318 (2)	0.0387 (6)	
C6	−0.18115 (7)	0.3592 (3)	0.0416 (2)	0.0479 (6)	
H6	−0.1838	0.4116	0.1207	0.058*	
C7	−0.21485 (7)	0.3765 (3)	−0.0651 (2)	0.0532 (7)	
H7	−0.2397	0.4428	−0.0589	0.064*	
C8	−0.21129 (8)	0.2955 (3)	−0.1788 (2)	0.0504 (7)	
C9	−0.17475 (8)	0.1981 (3)	−0.1915 (2)	0.0552 (7)	
H9	−0.1730	0.1419	−0.2696	0.066*	
C10	−0.14087 (7)	0.1860 (3)	−0.0857 (2)	0.0481 (6)	
H10	−0.1156	0.1232	−0.0938	0.058*	

### Atomic displacement parameters (Å^2^)

**Table:**

	*U*^11^	*U*^22^	*U*^33^	*U*^12^	*U*^13^	*U*^23^
K1	0.0449 (4)	0.0434 (5)	0.0523 (5)	0.000	0.0085 (3)	0.000
K2	0.0554 (5)	0.0446 (5)	0.0519 (5)	0.000	0.0097 (4)	0.000
Cl1	0.0570 (4)	0.1091 (7)	0.0574 (5)	−0.0104 (4)	−0.0191 (3)	0.0140 (4)
F1	0.0833 (10)	0.0700 (11)	0.0512 (9)	0.0116 (8)	0.0084 (8)	0.0191 (8)
F2	0.0436 (9)	0.1365 (16)	0.0512 (10)	0.0094 (8)	0.0132 (7)	0.0033 (9)
F3	0.0891 (11)	0.0680 (11)	0.0655 (10)	0.0008 (8)	0.0123 (8)	−0.0252 (9)
O1	0.0340 (9)	0.0642 (12)	0.0406 (9)	0.0020 (7)	0.0066 (7)	0.0005 (8)
O2	0.0340 (9)	0.0819 (13)	0.0434 (10)	0.0043 (7)	0.0013 (7)	−0.0037 (9)
O3	0.0436 (10)	0.0839 (14)	0.0388 (11)	0.0022 (9)	0.0063 (8)	−0.0031 (9)
C1	0.0429 (14)	0.0520 (16)	0.0430 (15)	0.0037 (11)	0.0034 (11)	−0.0001 (13)
C2	0.0368 (12)	0.0393 (14)	0.0408 (14)	0.0012 (9)	0.0055 (10)	−0.0001 (10)
C3	0.0309 (12)	0.0586 (16)	0.0402 (15)	0.0010 (10)	0.0050 (10)	0.0022 (12)
C4	0.0332 (12)	0.0410 (14)	0.0416 (15)	−0.0022 (9)	0.0035 (10)	0.0018 (11)
C5	0.0335 (12)	0.0437 (14)	0.0385 (14)	−0.0046 (9)	0.0047 (10)	0.0012 (11)
C6	0.0411 (13)	0.0571 (17)	0.0441 (15)	0.0034 (11)	0.0017 (11)	−0.0026 (12)
C7	0.0395 (13)	0.0629 (18)	0.0543 (17)	0.0059 (11)	−0.0018 (12)	0.0059 (14)
C8	0.0386 (14)	0.0657 (18)	0.0429 (16)	−0.0107 (12)	−0.0064 (11)	0.0128 (14)
C9	0.0522 (15)	0.0742 (19)	0.0384 (15)	−0.0107 (13)	0.0044 (12)	−0.0042 (13)
C10	0.0396 (13)	0.0591 (16)	0.0457 (16)	−0.0009 (11)	0.0064 (11)	−0.0006 (13)

### Geometric parameters (Å, º)

**Table:**

K1—O2^i^	2.7648 (17)	O1—C4	1.244 (3)
K1—O2^ii^	2.7648 (17)	O2—C2	1.266 (3)
K1—O3^iii^	2.832 (2)	O3—H1W	0.82 (3)
K1—O3	2.832 (2)	O3—H2W	0.87 (3)
K1—O1^i^	2.8640 (15)	C1—C2	1.518 (4)
K1—O1^ii^	2.8641 (15)	C2—C3	1.367 (3)
K1—F1^iii^	3.0415 (15)	C3—C4	1.419 (3)
K1—F1	3.0415 (15)	C3—H3	0.9300
K2—O3	2.7686 (19)	C4—C5	1.500 (3)
K2—O3^iii^	2.7686 (19)	C5—C10	1.376 (3)
K2—O2^iv^	2.7859 (17)	C5—C6	1.385 (3)
K2—O2^v^	2.7860 (17)	C6—C7	1.384 (3)
K2—O1^iv^	2.9457 (16)	C6—H6	0.9300
K2—O1^v^	2.9457 (16)	C7—C8	1.357 (3)
K2—F3^iii^	3.0692 (16)	C7—H7	0.9300
K2—F3	3.0692 (16)	C8—C9	1.376 (4)
Cl1—C8	1.744 (2)	C9—C10	1.378 (3)
F1—C1	1.336 (3)	C9—H9	0.9300
F2—C1	1.319 (3)	C10—H10	0.9300
F3—C1	1.334 (3)		
			
O2^i^—K1—O2^ii^	98.61 (7)	O2^iv^—K2—F3	110.86 (4)
O2^i^—K1—O3^iii^	164.81 (6)	O2^v^—K2—F3	97.85 (4)
O2^ii^—K1—O3^iii^	94.28 (5)	O1^iv^—K2—F3	162.20 (5)
O2^i^—K1—O3	94.29 (5)	O1^v^—K2—F3	61.73 (4)
O2^ii^—K1—O3	164.80 (5)	F3^iii^—K2—F3	136.07 (7)
O3^iii^—K1—O3	74.25 (8)	C1—F3—K2	142.49 (14)
O2^i^—K1—O1^i^	60.71 (5)	C4—O1—K1^i^	125.44 (14)
O2^ii^—K1—O1^i^	71.98 (5)	C4—O1—K2^iv^	114.54 (13)
O3^iii^—K1—O1^i^	116.65 (5)	K1^i^—O1—K2^iv^	77.52 (4)
O3—K1—O1^i^	121.86 (5)	C2—O2—K1^i^	123.39 (13)
O2^i^—K1—O1^ii^	71.98 (5)	C2—O2—K2^iv^	115.97 (13)
O2^ii^—K1—O1^ii^	60.71 (5)	K1^i^—O2—K2^iv^	81.90 (5)
O3^iii^—K1—O1^ii^	121.86 (5)	K2—O3—K1	104.74 (6)
O3—K1—O1^ii^	116.65 (5)	H1W—O3—H2W	99 (3)
O1^i^—K1—O1^ii^	104.48 (7)	F2—C1—F3	106.9 (2)
O2^i^—K1—F1^iii^	96.41 (4)	F2—C1—F1	106.7 (2)
O2^ii^—K1—F1^iii^	113.20 (4)	F3—C1—F1	104.6 (2)
O3^iii^—K1—F1^iii^	70.97 (5)	F2—C1—C2	115.3 (2)
O3—K1—F1^iii^	73.02 (5)	F3—C1—C2	111.0 (2)
O1^i^—K1—F1^iii^	60.63 (4)	F1—C1—C2	111.62 (19)
O1^ii^—K1—F1^iii^	164.83 (5)	O2—C2—C3	128.8 (2)
O2^i^—K1—F1	113.19 (4)	O2—C2—C1	113.20 (19)
O2^ii^—K1—F1	96.41 (4)	C3—C2—C1	118.0 (2)
O3^iii^—K1—F1	73.02 (5)	O2—C2—K2^iv^	45.21 (11)
O3—K1—F1	70.97 (5)	C3—C2—K2^iv^	95.70 (15)
O1^i^—K1—F1	164.83 (5)	C1—C2—K2^iv^	132.56 (14)
O1^ii^—K1—F1	60.63 (4)	C2—C3—C4	124.6 (2)
F1^iii^—K1—F1	134.38 (6)	C2—C3—H3	117.7
O3—K2—O3^iii^	76.27 (8)	C4—C3—H3	117.7
O3—K2—O2^iv^	93.79 (5)	O1—C4—C3	124.0 (2)
O3^iii^—K2—O2^iv^	165.95 (6)	O1—C4—C5	117.9 (2)
O3—K2—O2^v^	165.95 (6)	C3—C4—C5	118.12 (19)
O3^iii^—K2—O2^v^	93.79 (5)	C10—C5—C6	118.4 (2)
O2^iv^—K2—O2^v^	97.60 (7)	C10—C5—C4	119.5 (2)
O3—K2—O1^iv^	122.86 (5)	C6—C5—C4	122.1 (2)
O3^iii^—K2—O1^iv^	117.62 (5)	C7—C6—C5	120.7 (2)
O2^iv^—K2—O1^iv^	59.46 (5)	C7—C6—H6	119.7
O2^v^—K2—O1^iv^	70.46 (5)	C5—C6—H6	119.7
O3—K2—O1^v^	117.62 (5)	C8—C7—C6	119.2 (2)
O3^iii^—K2—O1^v^	122.86 (5)	C8—C7—H7	120.4
O2^iv^—K2—O1^v^	70.46 (5)	C6—C7—H7	120.4
O2^v^—K2—O1^v^	59.46 (5)	C7—C8—C9	121.8 (2)
O1^iv^—K2—O1^v^	100.47 (6)	C7—C8—Cl1	119.4 (2)
O3—K2—F3^iii^	75.48 (5)	C9—C8—Cl1	118.9 (2)
O3^iii^—K2—F3^iii^	70.27 (5)	C8—C9—C10	118.3 (2)
O2^iv^—K2—F3^iii^	97.85 (4)	C8—C9—H9	120.8
O2^v^—K2—F3^iii^	110.86 (5)	C10—C9—H9	120.8
O1^iv^—K2—F3^iii^	61.73 (4)	C5—C10—C9	121.6 (2)
O1^v^—K2—F3^iii^	162.20 (5)	C5—C10—H10	119.2
O3—K2—F3	70.26 (5)	C9—C10—H10	119.2
O3^iii^—K2—F3	75.48 (5)		
			
O2^i^—K1—K2—O3	13.40 (8)	O3^iii^—K1—F1—C1	74.8 (2)
O2^ii^—K1—K2—O3	−166.60 (8)	O3—K1—F1—C1	−3.9 (2)
O3^iii^—K1—K2—O3	180.0	O1^i^—K1—F1—C1	−153.4 (2)
O1^i^—K1—K2—O3	94.77 (8)	O1^ii^—K1—F1—C1	−141.6 (2)
O1^ii^—K1—K2—O3	−85.23 (8)	F1^iii^—K1—F1—C1	35.7 (2)
F1^iii^—K1—K2—O3	91.76 (7)	K2^vi^—K1—F1—C1	−144.3 (2)
F1—K1—K2—O3	−88.24 (7)	K2—K1—F1—C1	35.7 (2)
O2^i^—K1—K2—O3^iii^	−166.60 (8)	O3—K2—F3—C1	18.3 (2)
O2^ii^—K1—K2—O3^iii^	13.40 (8)	O3^iii^—K2—F3—C1	−62.1 (2)
O3—K1—K2—O3^iii^	179.998 (1)	O2^iv^—K2—F3—C1	104.8 (2)
O1^i^—K1—K2—O3^iii^	−85.23 (8)	O2^v^—K2—F3—C1	−154.0 (2)
O1^ii^—K1—K2—O3^iii^	94.77 (8)	O1^iv^—K2—F3—C1	158.5 (2)
F1^iii^—K1—K2—O3^iii^	−88.25 (7)	O1^v^—K2—F3—C1	157.1 (3)
F1—K1—K2—O3^iii^	91.75 (7)	F3^iii^—K2—F3—C1	−22.6 (2)
O2^i^—K1—K2—O2^iv^	0.0	C2^iv^—K2—F3—C1	89.0 (2)
O2^ii^—K1—K2—O2^iv^	180.0	C2^v^—K2—F3—C1	−150.4 (2)
O3^iii^—K1—K2—O2^iv^	166.60 (8)	K1^vii^—K2—F3—C1	157.4 (2)
O3—K1—K2—O2^iv^	−13.40 (8)	K1—K2—F3—C1	−22.6 (2)
O1^i^—K1—K2—O2^iv^	81.37 (6)	O3^iii^—K2—O3—K1	0.0
O1^ii^—K1—K2—O2^iv^	−98.63 (6)	O2^iv^—K2—O3—K1	169.93 (6)
F1^iii^—K1—K2—O2^iv^	78.35 (5)	O2^v^—K2—O3—K1	−45.9 (2)
F1—K1—K2—O2^iv^	−101.65 (5)	O1^iv^—K2—O3—K1	114.23 (6)
O2^i^—K1—K2—O2^v^	180.0	O1^v^—K2—O3—K1	−120.17 (5)
O2^ii^—K1—K2—O2^v^	0.0	F3^iii^—K2—O3—K1	72.79 (6)
O3^iii^—K1—K2—O2^v^	−13.40 (8)	F3—K2—O3—K1	−79.25 (6)
O3—K1—K2—O2^v^	166.60 (8)	C2^iv^—K2—O3—K1	152.89 (7)
O1^i^—K1—K2—O2^v^	−98.63 (6)	C2^v^—K2—O3—K1	−58.15 (12)
O1^ii^—K1—K2—O2^v^	81.36 (6)	K1^vii^—K2—O3—K1	180.0
F1^iii^—K1—K2—O2^v^	−101.65 (5)	O2^i^—K1—O3—K2	−169.85 (6)
F1—K1—K2—O2^v^	78.35 (5)	O2^ii^—K1—O3—K2	42.1 (2)
O2^i^—K1—K2—O1^iv^	−81.37 (6)	O3^iii^—K1—O3—K2	0.0
O2^ii^—K1—K2—O1^iv^	98.63 (6)	O1^i^—K1—O3—K2	−111.93 (6)
O3^iii^—K1—K2—O1^iv^	85.23 (8)	O1^ii^—K1—O3—K2	118.17 (6)
O3—K1—K2—O1^iv^	−94.77 (8)	F1^iii^—K1—O3—K2	−74.44 (6)
O1^i^—K1—K2—O1^iv^	0.0	F1—K1—O3—K2	77.08 (6)
O1^ii^—K1—K2—O1^iv^	179.999 (1)	K2^vi^—K1—O3—K2	180.0
F1^iii^—K1—K2—O1^iv^	−3.01 (5)	K2—F3—C1—F2	155.52 (15)
F1—K1—K2—O1^iv^	176.99 (5)	K2—F3—C1—F1	42.6 (3)
O2^i^—K1—K2—O1^v^	98.63 (6)	K2—F3—C1—C2	−78.0 (3)
O2^ii^—K1—K2—O1^v^	−81.36 (6)	K1—F1—C1—F2	−165.34 (14)
O3^iii^—K1—K2—O1^v^	−94.77 (8)	K1—F1—C1—F3	−52.3 (3)
O3—K1—K2—O1^v^	85.23 (8)	K1—F1—C1—C2	67.8 (3)
O1^i^—K1—K2—O1^v^	180.0	K1^i^—O2—C2—C3	−45.8 (3)
O1^ii^—K1—K2—O1^v^	0.0	K2^iv^—O2—C2—C3	51.9 (3)
F1^iii^—K1—K2—O1^v^	176.99 (5)	K1^i^—O2—C2—C1	134.65 (16)
F1—K1—K2—O1^v^	−3.01 (5)	K2^iv^—O2—C2—C1	−127.69 (16)
O2^i^—K1—K2—F3^iii^	−80.95 (5)	K1^i^—O2—C2—K2^iv^	−97.66 (15)
O2^ii^—K1—K2—F3^iii^	99.05 (5)	F2—C1—C2—O2	−175.63 (19)
O3^iii^—K1—K2—F3^iii^	85.65 (7)	F3—C1—C2—O2	62.7 (3)
O3—K1—K2—F3^iii^	−94.36 (7)	F1—C1—C2—O2	−53.6 (3)
O1^i^—K1—K2—F3^iii^	0.41 (5)	F2—C1—C2—C3	4.7 (3)
O1^ii^—K1—K2—F3^iii^	−179.59 (5)	F3—C1—C2—C3	−116.9 (2)
F1^iii^—K1—K2—F3^iii^	−2.60 (4)	F1—C1—C2—C3	126.8 (2)
F1—K1—K2—F3^iii^	177.40 (4)	F2—C1—C2—K2^iv^	134.69 (17)
O2^i^—K1—K2—F3	99.05 (5)	F3—C1—C2—K2^iv^	13.0 (3)
O2^ii^—K1—K2—F3	−80.95 (5)	F1—C1—C2—K2^iv^	−103.3 (2)
O3^iii^—K1—K2—F3	−94.35 (7)	O2—C2—C3—C4	−0.1 (4)
O3—K1—K2—F3	85.64 (7)	C1—C2—C3—C4	179.5 (2)
O1^i^—K1—K2—F3	−179.59 (5)	K2^iv^—C2—C3—C4	34.1 (2)
O1^ii^—K1—K2—F3	0.41 (5)	K1^i^—O1—C4—C3	40.2 (3)
F1^iii^—K1—K2—F3	177.40 (4)	K2^iv^—O1—C4—C3	−51.7 (2)
F1—K1—K2—F3	−2.60 (4)	K1^i^—O1—C4—C5	−139.83 (15)
O2^i^—K1—K2—C2^iv^	−18.02 (6)	K2^iv^—O1—C4—C5	128.26 (16)
O2^ii^—K1—K2—C2^iv^	161.98 (6)	C2—C3—C4—O1	2.5 (4)
O3^iii^—K1—K2—C2^iv^	148.58 (8)	C2—C3—C4—C5	−177.4 (2)
O3—K1—K2—C2^iv^	−31.42 (8)	O1—C4—C5—C10	30.2 (3)
O1^i^—K1—K2—C2^iv^	63.35 (6)	C3—C4—C5—C10	−149.8 (2)
O1^ii^—K1—K2—C2^iv^	−116.65 (6)	O1—C4—C5—C6	−148.3 (2)
F1^iii^—K1—K2—C2^iv^	60.33 (5)	C3—C4—C5—C6	31.6 (3)
F1—K1—K2—C2^iv^	−119.67 (5)	C10—C5—C6—C7	−1.1 (3)
O2^i^—K1—K2—C2^v^	161.98 (6)	C4—C5—C6—C7	177.5 (2)
O2^ii^—K1—K2—C2^v^	−18.02 (6)	C5—C6—C7—C8	1.9 (4)
O3^iii^—K1—K2—C2^v^	−31.42 (8)	C6—C7—C8—C9	−0.8 (4)
O3—K1—K2—C2^v^	148.58 (8)	C6—C7—C8—Cl1	178.80 (19)
O1^i^—K1—K2—C2^v^	−116.65 (6)	C7—C8—C9—C10	−1.1 (4)
O1^ii^—K1—K2—C2^v^	63.35 (6)	Cl1—C8—C9—C10	179.34 (18)
F1^iii^—K1—K2—C2^v^	−119.67 (5)	C6—C5—C10—C9	−0.8 (4)
F1—K1—K2—C2^v^	60.33 (5)	C4—C5—C10—C9	−179.5 (2)
O2^i^—K1—F1—C1	−90.4 (2)	C8—C9—C10—C5	1.9 (4)
O2^ii^—K1—F1—C1	167.4 (2)		

Symmetry codes: (i) −*x*, −*y*, −*z*+1; (ii) *x*, −*y*, *z*+1/2; (iii) −*x*, *y*, −*z*+3/2; (iv) −*x*, −*y*+1, −*z*+1; (v) *x*, −*y*+1, *z*+1/2; (vi) *x*, *y*−1, *z*; (vii) *x*, *y*+1, *z*.

### Hydrogen-bond geometry (Å, º)

**Table:**

*D*—H···*A*	*D*—H	H···*A*	*D*···*A*	*D*—H···*A*
O3—H1*W*···O2	0.82 (3)	1.90 (3)	2.709 (2)	173 (3)
O3—H2*W*···O1^viii^	0.87 (3)	2.06 (3)	2.843 (3)	150 (2)

Symmetry code: (viii) −*x*, *y*, −*z*+1/2.

## References

[bb1] Bruker (2003). *SADABS*, *SMART* and *SAINT* Bruker AXS Inc., Madison, Wisconsin, USA.

[bb2] Johnson, C. K. (1976). *ORTEPII* Report ORNL-5138. Oak Ridge National Laboratory, Tennessee, USA.

[bb3] Martín-Ramos, P., Coya, C., Alvarez, A. L., Ramos Silva, M., Zaldo, C., Paixão, J. A., Chamorro-Posada, P. & Martín-Gil, J. (2013*a*). *J. Phys. Chem. C*, **117**, 10020–10030.

[bb4] Martín-Ramos, P., Ramos-Silva, M., Coya, C., Zaldo, C., Alvarez, A. L., Alvarez-García, S., Matos-Beja, A. M. & Martín-Gil, J. (2013*b*). *J. Mater. Chem. C*, **1**, 2725–2734.

[bb5] Sheldrick, G. M. (2008). *Acta Cryst.* A**64**, 112–122. 10.1107/S0108767307043930 18156677

